# Public Health Ambassadors: A Novel Participatory Community Health Awareness Program in Abu Dhabi

**DOI:** 10.1177/00469580241241268

**Published:** 2024-09-14

**Authors:** Iffat Elbarazi, Jamila Al-Balushi, Aisha Al-Dhaheri, Mumtaz Meeran, Mariana Peyroteo, Marília Silva Paulo, Michal Grivna

**Affiliations:** 1 Institute of Public Health, College of Medicine and Health Sciences, United Arab Emirates University, Al Ain, United Arab Emirates; 2 Abu Dhabi Public Health Center, Abu Dhabi, United Arab Emirates; 3 CHRC, NOVA Medical School, Faculdade de Ciências Médicas, NMS, FCM, Universidade Nova de Lisboa, Lisboa, Portugal

**Keywords:** Public health ambassadors, community-based interventions (CBI), intervention mapping, community participation, social mobilization

## Abstract

Community-based intervention (CBI) programs promote lifestyle changes, modify risk factors, and substantially improve public health. Social mobilization and community involvement improve health outcomes, reduce health disparities, and improve access to care and services. Health intervention program evaluations are essential to provide evidence-based strategies that can enhance the design and implementation of successful health promotion programs. Interventions that enable the United Arab Emirates (UAE) community to change and modify unhealthy behaviors were the priority of the last decade and are the health authorities’ objectives. The Department of Health Abu Dhabi launched a wellness program to enable the community to adopt healthy behaviors. The Public Health Ambassadors program is a community-based health intervention program under the Abu Dhabi Public Health Centre, inaugurated in 2019. This paper describes the Public Health Ambassadors CBI conducted in Abu Dhabi. The implementation science framework was used to develop the intervention. The Public Health Ambassadors is one of the UAE’s earliest and most successful CBIs. The program can be used as a model to encourage more health promotion interventions in the country and the region. The role of the program was highlighted during the COVID-19 pandemic. Voluntary community participation and social responsibilities are essential competencies promoted by this program.

**What do we already know about this topic?**
Community health promotion programs and interventions programs encourage lifestyle changes, modify risk factors, and have a high impact on population’s health. Evidence-informed health interventions are based on a theoretical framework that involves different community groups and stakeholders, resulting in more effective strategies and interventions.
**What is the Public Health Ambassador program?**
The Public Health Ambassador program is a community-based health intervention program aligned with the global commitment to good health and well-being. This program is the first in the country and it is aligned with the UAE’s Sustainable Development Goals.
**How this program contributes to improve population’s health?**
This interventional community program is based on the training of active community members who have exhibited an interest in promoting health. The program teaches the ambassadors about the public health priorities, determinants, and risk factors in the UAE and healthy lifestyle guidelines, including nutrition, smoking cessation, stress management, exercise, and mental health.
In addition to embassies has assisted us in reaching different segments of the society and encourage community members to lead healthy lifestyles through peers.

## Background

Community health promotion programs and community-based intervention (CBI) programs are known to promote lifestyle changes, modify risk factors, and have a vast public health impact.^1^ Evidence-informed health intervention planning and implementation are based on a theoretical framework that engages different community groups and stakeholders, resulting in more effective strategies and interventions.^2^ These principles, like community participation, civic engagement, and community mobilization in health care, are basic principles that emerged with the Alma Ata Declaration in 1972 and are still valid nowadays.^3^ Social mobilization is one of the main pillars of health promotion.^4^ Community involvement and its participation have shown a positive impact in reducing health disparities, improving access to care and services,^5,6^ and improving health outcomes in fields such as maternal and child health, HIV, childhood immunization, and diabetes.^7-9^ Moreover, community participation and social mobilization are essential dimensions of the social determinants of health framework actions.^10^ Studies and action research methodology have used community participation to improve the success of interventions and health awareness among community sectors.^11^ Health intervention program evaluations are essential to provide evidence-based strategies that enhance the design and implementation of successful health promotion programs.^12^ Previous studies found that such assessments influence health policies and behavioral change, improving the success of intervention programs.^13^ Additionally, some studies found that improvement of the programs influences specific health and long-term health outcomes, such as mortality.^14^ Longitudinal studies are needed to evaluate interventional and community improvement programs, and such studies should evaluate the threats and barriers to the success of such programs and how to avoid them.^12^

Guidance on the concept and design of health promotion programs is crucial to identify strategies to improve the program’s efficacy, allocate resources, and identify ways to improve practice and outcomes.^15,16^ Implementation Science (IS) uses a systematic approach to plan interventions, aims to improve intervention quality, effectiveness, and sustainability, and allows the translation of proven interventions into effective real-world interventions.^17^ It comprises a set of frameworks and follows a stepwise process with 3 phases: pre-implementation (diagnosis), implementation (intervention), and post-implementation (evaluation). A model proposed by Aarons et al that articulates the variables in community-based participatory action research that plays an important role in the effective implementation of evidence-based practices highlights the 4 phases of implementation (Exploration, Preparation, Implementation, Sustainment—EPIS) and the different aspects of the external and internal context that may be more prominent or manifest differently during different phases.^18^

The United Arab Emirates (UAE) is a fast-developing country located in the Arabian Peninsula. It is a federation of 7 Emirates (Abu Dhabi, Ajman, Dubai, Fujairah, Ras Al Khaimah, Sharjah, and Umm Al-Quwain) formed in 1971. Its population in the year 2019 was around 9.7 million.^19^ Approximately 11.4% of this population are Emirati citizens, while the rest are expatriates. Abu Dhabi is the UAE’s capital, comprising around 34.7% of the population. Islam is the official religion, and Arabic is the official language. The political system in UAE is a monarchy where every of the 7 Emirates is governed by a ruler (Sheikh). The federal supreme council elects the president and the minister for the federal government. The health system in the UAE is a government-funded health service with a growing private sector that provides secondary and tertiary care. Different authorities regulate healthcare at the Federal and Emirate levels: the Ministry of Health and Prevention, the Department of Health Abu Dhabi (previously called Health Authority-Abu Dhabi), the Dubai Health Authority, the Dubai Healthcare City, and the Sharjah Health Authority. The Abu Dhabi Public Health Centre (ADPHC) was launched in 2019 to ensure a public health system that maintains the population’s health in the Emirate and guarantees workers’ safety through the promotion of public health and preventive health concepts. The ADPHC is the first of its kind in the region.

Significant public health challenges in the Emirate of Abu Dhabi include obesity, diabetes, and cardiovascular diseases. These challenges include unhealthy diets, physical inactivity, tobacco consumption, a sedentary lifestyle, and injuries. As per the Department of Health Abu Dhabi, heart disease is the leading cause of death in the emirate, accounting for 37% of all fatalities in 2016.^19^ In addition to having a high prevalence of diabetes, only 31% of adults report being physically active, and a high proportion of children are obese in the Emirate.^20^ Interventions that enable the UAE community to change and modify these unhealthy behaviors were set as priorities for the last decade and are one of the critical targets and objectives of health authorities in the UAE. The current priorities defined by the Department of Health Abu Dhabi are keeping the population healthy mentally and physically, a regional leader in care excellence, a financially sustainable healthcare system, a strong home-grown healthcare workforce, a regional leader in life science research, globally leading digital healthcare ecosystem, and robust governance of the healthcare sector. The UAE has its own Sustainable Development Goals (SDGs) set to meet the global commitment, and one of the activities assigned to meet some of the UAE targets is the Public Health Ambassador program (PHAP) (SDG 3 & 17).^21,22^

In the present study, based on the implementation science, we aim to describe the Public Health Ambassadors CBI conducted in Abu Dhabi by its health authority.

## Methods

In 2015, the Department of Health Abu Dhabi launched a wellness program to enable the community to adopt healthy behaviors. The Public Health Ambassador program is a community-based health intervention program aligned with the global commitment to good health and well-being. Based on the Implementation Science frameworks, the EPIS Framework was used for the development of this intervention and involved answering 3 questions: “*when*,” “*who*,” and “*what*.”^17,18^ According to Villalobos Dintrans et al,^17^ an intervention can be described as time-based or component-based.

- **Time-based**—describing the intervention according to time involves 6 stages (diagnosis, planning, action, monitoring, evaluation, and change).
- **Component-based**—description of the intervention according to the actors involved (providers, recipients) and according to the external elements involved.

The program is described in the results section following this methodology. As the intervention is ongoing, there is no description of the results of the post-implementation evaluation.

## Results of the Public Health Ambassadors Program

### Time-Based Framework

#### Diagnosis

The project coordinators conducted a rapid appraisal to justify the importance of such a program. An essential perceived need was that community participation in preventive programs still needed to be improved in the UAE. Despite extensive efforts by the relevant agencies, community engagement and uptake of preventative programs such as comprehensive screening, regular dental checkups, and routine vaccination remained low. Unhealthy lifestyle behaviors are widespread, including physical inactivity, unhealthy diets, and tobacco consumption. Based on these findings, the program was recognized as a need and adopted the one-to-one communication strategy as an effective health education strategy to enhance health promotion, enable individuals to adopt healthier lifestyles, and engage them with health preventative services. One-to-one education has a solid and clear impact on convincing individuals of the need for change.

#### Planning

Considering the identified needs, the purpose of the PHAP is to emphasize the role of community engagement, empowerment, and participation in disseminating Public Health messages in the Emirate of Abu Dhabi to promote the health of individuals and encourage them to adopt healthier lifestyle practices. The program’s specific objectives are to increase awareness about public health priorities to encourage community members to follow a healthy lifestyle, increase access to public health programs and preventive services for individuals and communities, and develop a network of Public Health Ambassadors in various regions of Abu Dhabi.

To this end, the strategy defined was based on all ambassadors participating in a 5-day training program covering general topics related to health and work and services offered by ADPHC (Table 1). On completion of training, Public Health Ambassadors are certified by the ADPHC, and the certification is valid for 2 years and it is renewable. The specific requirements for certification and renewal are:

**Table 1.** Public Health Ambassadors Training Content.

Overview of the program and its objectives.
Basic information: health, health promotion and awareness.
Public health priorities and challenges.
Discussing public health issues affecting Abu Dhabi: overview of each problem and ways to tackle each problem.
Recommend lifestyle practices and behaviors.
The importance of understanding these behaviors and their implementation to health.

- Full attendance at the Public Health Ambassadors training program.
- Attendance at all other training and continuing education workshops.
- Attendance at periodic meetings.
- Submission of biannual reports on activities undertaken in the community.
- Presentation of an annual report highlighting achievements and success stories.
- Generation of new ideas and projects that are beneficial to community members to increase awareness among them and to adopt healthy lifestyles.

Role of the Ambassadors in Health Promotion:

- Educate society about adopting health practices, influence their peers, and motivate them to start the change toward healthier lifestyles that help prevent diseases in the future.
- Strengthen the will of individuals to adopt healthier practices and support the culture of change in enabling community members to adopt a healthy lifestyle.
- Spread awareness about all diseases that may be prevented and disseminate information on the importance and methods of early disease detection and diagnosis.
- Disseminate information on health and services initiated and announced by ADPHC commensurate with their environment and peers.
- Participate in awareness campaigns and external activities held by ADPHC and support its programs.
- Understand the developed methods and techniques to provide such information meaningfully, depending on all targeted people and individual segments.

### Acting

This interventional community program is based on the training of active community members who have exhibited an interest in promoting health. The training course is designed to meet the needs of the trainers and the community. The course covers the basic definition of health concepts and healthy individuals, health promotion, and awareness, followed by public health priorities and challenges in the country, social determinants of health, and human behavior change concepts. Participants must complete a pre-training survey to assess their baseline knowledge and understanding of the subject matter at the start of the training course. The participants are presented with specific educational materials and invited to discuss the public health issues in Abu Dhabi, how to tackle each problem and recommend lifestyle practices and behaviors. The essential qualities and skills expected from Public Health Ambassadors, such as core skills for communicating health messages and interacting with the community, are also addressed in the training. The sessions are delivered in a lecture format and offered interactively and engagingly. The sessions provide ample opportunities for participants to interact with the trainer and other attendees to learn from each other. All ambassadors are rewarded with issued certificates and directed to provide community awareness activities organized by the Ambassadors’ program director on the topics covered in the course (Table 1).

Between 2022 and 2023, more than 107 workshops were conducted, with a number of beneficiaries between 60,000 and 53,000 at each and the number of beneficiaries through social media reaching around 40,000 people from the UAE community.

### Monitoring and Continuous Evaluation

During specific health awareness/promotion events, daily debriefing meetings take place, and reports are provided. One-to-one and group discussions also happen after they participate in any event, and verbal reports are collected. For example, during their participation at the Sheikh Zayed Festival, ambassadors gave immediate feedback, pointed to some challenges, and needed actions that led to better program delivery.

After completing the training, the program includes regular communication and training to Ambassadors with meetings every 2 months or 3 months in each region (Abu Dhabi, Al Ain, or Al Dhafra) or online. Moreover, each year, all the Public Health Ambassadors are invited for professional development course, with reinforcement of contents and methods for community awareness activities.

There is also an instant message group (WhatsApp) to discuss inquiries or topics that interest the ambassadors. The group also uses Microsoft TEAMS for urgent tasks or to clarify the doubts requested from the ambassadors.

As mentioned above, all participants are required to complete a pre-training questionnaire to assess their baseline knowledge and understanding of public health topics and communication methods. After training completion, the same questionnaire is administered to assess improvements from the baseline. It was noted that the time required to complete the questionnaire has significantly reduced following training exercises, and modifications in scores were noted.

An evaluation is undertaken at the end of each training day. Participants evaluate the trainers and provide their feedback on the day. Also, the ambassadors themselves provide their evaluation and feedback. This is an important task needed to make any alterations required for the following day.

The ambassadors’ training evaluation is ongoing. The training is conducted through workshops conducted over 3 to 5 days. During the workshops, the ambassadors’ information is refreshed. The training also ensures to cover their education and communication and skills. Moreover, during the training, the ambassadors can provide feedback and discuss their challenges and strategies to improve the program.

For example, in a workshop conducted in November 2021, the Public Health Ambassadors provided a list of challenges they faced. The list included the topics of difficulty moving from cities, lack of belief or appreciation from external parties in the role of ambassadors, absence of an agreement between the center and the administration, and short notice for the health promotion events. These activities and feedback are part of the process evaluation of the program. In this manuscript, we do not discuss the impact and outcome evaluation of the program as this program is continuous. Impact evaluation is planned soon, and once conducted, it will be presented later.

Due to the nature of the program and the implementation of public health strategies into the community, the program has been running smoothly and there is no outcome assessment evaluation yet. But it is planned in to be implemented in the upcoming year as the program is reaching is full capacity and there is a need to evaluate its effectiveness and current challenges, such as the voluntary basis of the participants. Although there is no formal evaluation, the impact on economic and social level is reflected. The PHAP has contributed to saving money (cost of manpower, venue, time, resources) thereby adding to revenue of AD government. Socially, this culturally sensitive training course is delivered by local experts in line with UAE culture and norms, making the program more effective for adoption by target audience.

### Changes

The Public Health Ambassadors is a health initiative that has 2 primary outcomes. Community participation and action and raising community awareness about public health actions and prevention are the primary outcomes of this innovative program in Abu Dhabi. The program is unique in the UAE and the region. It is a new initiative that encourages community participation and adds to the actions taken by the government to empower communities and stimulate social responsibility.

The ADPHC launched the PHAP in 2015 to empower the community on key public health themes. After establishing the ADPHC in 2019,^23^ the Public Health Ambassadors became part of the health promotion section operating under the center. This interventional community health program focuses on training people who support adopting healthier lifestyles to influence their peers and encourage them to change toward a healthy lifestyle. Public Health Ambassadors are neither advisors nor guides, but community members from different occupations contribute to disseminating health information and services undertaken and announced by the ADPHC commensurate with their environment and peers. They inspire and stimulate their peers to adopt and enjoy a healthier lifestyle by engaging in physical activity and periodic health checkups. They are voluntary and do not receive any monetary compensation.

The Public Health Ambassadors’ contribution to maintaining the population’s health and well-being was evident during the pandemic. Dependent on the stages of the pandemic, the ambassadors were critical in supporting the pandemic containment efforts, including raising awareness of COVID-19, the importance of adhering to the preventive measures, providing evidence-based information on vaccination, ensuring health and safety at different events (once this was allowed in the Emirate). Similarly, the topics and communication tools are constantly updated based on the UAE public health priorities and community health challenges.

One of the critical areas of work for the ambassadors was convincing individuals aged 50 and above who were eligible but chose not to receive the vaccine as part of the “We reach you wherever you are” initiative. Through an IT platform established for this initiative, the ambassadors received details of eligible individuals with whom they set one-to-one contact and convinced them to receive the vaccine by alleviating their fears and concerns by providing evidence-based information and dispelling rumors and myths. Additional information was provided to those not persuaded to enable them to receive the vaccine. Most recent data shows that around 40% of individuals contacted by the ambassadors were influenced to take the vaccine.

### Component-Based Framework

#### Receivers

The Ambassadors are usually recruited from their workplace; preferably, they should be from the Health and Safety Department, although this is not mandatory and it is based on voluntary work from the Ambassadors. Participants are nominated to enroll in the program by their employer based on the eligibility of the inclusion criteria. After their nomination, each participant completes an application form stating how their experience and skills match the requirements to be a Public Health Ambassador. The ADPHC team reviews all the applications, and only participants demonstrating their expertise and skills are selected for the training program. The skills assessed and considered as eligibility criteria focus on the interest in learning and understanding health-related issues, their demonstrated commitment to the program, their enthusiasm, their communication and presentation skills, and their social media use and behavior (Table 2).

**Table 2.** Eligibility Criteria for Public Health Ambassadors.

● The essential qualities and skills that public health ambassadors’ needs:
● Demonstrable interest in learning and understanding health and health-related issues.
● Committed to continuous personal and professional development.
● Enthusiastic regarding gaining new skills.
● Social media presence—social channels of communication with 100 persons or above.
● Excellent communication and presentation skills—dissemination of basic information related to the Department of Health Programs.
● Excellent organizational skills—organize community activities to raise awareness on programs expressed at the course and any other health matters.
● Support and organize similar awareness programs, if any.

#### Public Health Ambassadors in the community

There are 249 Public Health Ambassadors in Abu Dhabi covering the 3 regions—Al Ain, Abu Dhabi, and Al Dhafra to ensure equity of service provision. Since the program’s inception in 2015, one batch per year has received training, and 6 training courses have been conducted. The minimum number of participants per training course was 19, and the maximum was 45. The number of Ambassadors has grown significantly since program inception, expanding from 25 in 2015 to 249 in 2023, representing 65 government and private sector entities (including educational and health sectors). The majority of Ambassadors are from Emirati nationality followed by Arabic nationalities, only 49 were trained in English are form non-Arabic speaking countries.

Upon completion of training and certification, the ambassadors carry out health-promoting activities in their communities with their peers, dependent on needs and as guided by ADPHC. Ambassadors also play a crucial role in ensuring health and safety at many events. Moreover, peers (friends or relatives) are crucial in affecting the individual’s decision-making and supporting behavior change. Developed countries are investing in promoting the health of individuals through “Health Ambassador” or “Peer to Peer” programs, using several volunteers equipped with information on health and local services and possessing positive communication skills to encourage their peers to adopt a healthier lifestyle.^24^

## Future Direction

Future plans include continuing the program and recruiting more eligible volunteers, officially registering the program in the UAE, and having award ceremonies for accredited Ambassadors. Developing an evaluation framework for the program in collaboration with academic institutions is the next step of the Public Health Ambassadors program with the implementation of an effectiveness evaluation model. The development of a PHAP IT platform is also currently being developed by UAE corporate communications sector.

## Conclusion

There were no other existing programs in the UAE that involved volunteers in health education until the initiation of this program. The Public Health Ambassadors represent an effective communication channel to disseminate information in the community sustainably and use low financial resources. Moreover, the program teaches the ambassadors about the public health priorities, determinants, and risk factors in the UAE and healthy lifestyle guidelines, including nutrition, smoking cessation, stress management, exercise, and mental health. We recommend community based-interventions such as this program to be implemented, and the Public Health Ambassadors can be adapted as role models to be implemented and followed in other countries.
